# Efficacy and safety of topical glycopyrronium bromide in treating axillary hyperhidrosis: systematic review and meta-analysis

**DOI:** 10.1038/s41598-024-74430-4

**Published:** 2024-10-19

**Authors:** Amr Elrosasy, Mohamed Abo Zeid, Raghad Samha, Hazim Alkousheh, Shirin cadri, Nina cadri, Doaa Mashaly, Aya Ehab, Lava Abdullah, Esraa M. AlEdani

**Affiliations:** 1 Faculty of Medicine, Cairo University, Cairo, Egypt; 2 Faculty of Medicine, Tanta University, Tanta, Egypt; 3 Faculty of Medicine, AlBaath University, Homs, Syrian Arab Republic; 4 Faculty of Medicine, Hashemite University, Zarqa, Jordan; 5 University of Medicine and Pharmacy Grigore T. Popa, Iași, Romania; 6 Faculty of medicine, October 6 university, 6th of October City, Egypt; 7 Faculty of Medicine, Aswan University, Aswan, Egypt; 8 Zaira Research Academy, Damascus, Syrian Arab Republic; 9 Basra Medical College, Basra, Iraq

**Keywords:** Hyperhidrosis, Glycopyrronium Bromide, Meta-analysis, Quality of life, Adverse events, Drug discovery, Skin diseases

## Abstract

**Background:**

Hyperhidrosis (HH), characterized by excessive sweating, poses a significant challenge to patients’ quality of life. This meta-analysis evaluates the safety and efficacy of topical glycopyrronium bromide (GBP) in treating primary hyperhidrosis, a chronic condition affecting various body regions. Despite its prevalence, primary axillary hyperhidrosis is often undertreated due to a lack of awareness and social stigma.

**Methods:**

Following PRISMA guidelines, we conducted a systematic review and meta-analysis of randomized controlled trials comparing GBP to a placebo in primary hyperhidrosis patients. Eligibility criteria included outcomes related to perspiration suppression and symptom improvement.

**Results:**

Four RCTs involving 1401 patients were included. GBP significantly increased Hyperhidrosis Disease Severity Scale (HDSS) responders (RR = 2.33, 95% CI [1.99 to 2.74], *p* < 0.00001) and Axillary Sweating Daily Diary (ASDD/ASDD-C) responders (MD = 3.07, 95% CI [2.32 to 4.06], *p* < 0.002) without significantly causing adverse events. Dermatology life quality index was also significantly improved in the GBP group (MD = -2.32, 95% CI [-3.09, -1.55], *P* < 0.00001).

**Conclusion:**

GBP demonstrated effectiveness in reducing sweat production while improving HDSS and DLQI scores. Adverse events included dry mouth and anticholinergic effects. Dry eye and local skin reactions were not significant, which makes GBP promising in managing primary hyperhidrosis, offering improvements in symptoms and quality of life. While adverse events should be considered, further research with larger sample sizes and long-term follow-up is warranted for comprehensive clinical integration.

## Key points

• We found that topical glycopyrronium can be an effective treatment for hyperhidrosis, reducing sweat production and improving patients’ symptoms.

• We also found that the increase in certain adverse events necessitates careful consideration in clinical decision-making.

## Introduction

Hyperhidrosis (HH) is characterized by excessive and disproportionate sweating beyond what is expected to control body temperature. It’s a chronic condition and can be classified into primary and secondary forms^1,2^. Statistically, the prevalence of primary HH in the US is 4.8%, 12.8% in Japan, and 16.3% in Germany^1,3^.

Primary hyperhidrosis is described as idiopathic and affects specific body regions, usually the axillae, palms, soles, or craniofacial region, resulting from overactive sympathetic nerves. Primary axillary hyperhidrosis is characterized by excessive underarms sweating and bilateral presentation^4,5^. Secondary HH could be due to certain medical conditions (endocrine, neurological, cardiovascular, infection and neoplasm) or pharmacological causes^2,6^.

The adverse effects of HH on life quality could be anxiety and depression, which lead to decreased work productivity and could be more than 3.5 times more prevalent in patients with HH. In addition, low self-compassion and imposter phenomenon may affect the mental, social, professional, and educational domains of lives in patients with HH^3,4,7–10^. Most patients with primary axillary hyperhidrosis either wait a few years to seek medical attention or do not seek treatment at all, despite the condition’s high prevalence rates, severe symptoms, and impact on quality of life. This is probably due to a lack of awareness that primary axillary hyperhidrosis is treatable, treatment compliance, or due to social stigma of excessive sweating and low level of satisfaction^3,4,11–13^.

Treatments for hyperhidrosis have generally involved blocking sweat from reaching the skin’s surface (e.g., antiperspirants) containing aluminium salts in range from 10 to 35%, blocking neuronal transduction to the sweat glands (e.g., onabotulinumtoxin A or oral anticholinergic drugs), destroying the sweat glands (e.g., thermal ablation or surgical removal) or using electrical current to suppress excessive sweating (e.g., iontophoresis)^1,2,14,15^. Anticholinergic substances like Glycopyrronium tosylate (GT) functions as a competitive inhibitor of acetylcholine receptors and can be used as an oral or topical treatment of HH. Oral use is associated with reductions in clinical symptoms and improved quality of life, but at the expense of significant systemic adverse events. While topical formulations containing oxybutynin, umeclidinium, or sofpironium have been used successfully to treat HH, they are not yet approved for clinical use in the European Union. In 2018, the US Food and Drug Administration (FDA) approved GT 3.75% as a topical anticholinergic medication for patients ≥ 9 years old with primary axillary hyperhidrosis^1,14,16^.

Because HH treatments are administered via a variety of routes and methods, patients must have reliable information to help them make the most appropriate choice for their individual needs. It’s important to investigate the safety and efficacy of Glycopyrronium in treatment of primary hyperhidrosis, which was the aim of this meta-analysis.

## Methods

We followed the guidelines of the Preferred Reporting Items for Systematic Reviews and Meta-analysis (PRISMA) in conducting this systematic review and meta-analysis^17^.

### Eligibility criteria and study selection

We included studies accordingly if they matched the inclusion criteria: (a) Randomized-controlled trials that used glycopyrronium bromide as an intervention against placebo, (b) Quantitative suppression of perspiration, (c) Utilizing HDSS, and (d) Utilizing Dermatology life quality index (DLQI). We excluded case reports, case series, animal studies, laboratory, editorials, non- English literature, unpublished literatures, reviews, thesis, conference abstracts and studies that didn’t mention glycopyrronium bromide as a treatment for patients with hyperhidrosis.

### Search strategy

We searched electronically through Pubmed, Web of Science, Scopus, Embase, MedLine and ovid to identify the relevant available RCTs from inception till April 2024, using the following search strategy (“Glycopyrronium Bromide” OR Pyrrolidinium OR NVA237 OR Glycopyrrolate) AND (hyperhidrosis). After removing the duplicate studies, title and abstract screening were done using Rayyan software package^18^, then full text screening of included abstracts was done to finalise the included studies.

### Statistical analysis

The statistical analysis was conducted using RevMan Cochrane. The continuous variables were described using mean and standard deviation (SD), while the categorical variables were described using numbers and percentages^19^.

### Data extraction

We used a uniform data extraction sheet that was pre-specified to extract the data from each included study and two independent authors participated in it. The extracted sheet includes the summary which contains (intervention, study design, country, numbers of centers, total participants, duration of treatment, main inclusion criteria, primary outcomes, conclusion, and list of ingredients in study drugs), baseline characteristics, and the efficacy outcome.

### Quality assessment

We implemented a Cochrane risk-of-bias tool for randomised control trials (RoB 2)^20^, which contain five domains: Randomization process (bias in selection) of all reported results, deviation from intended intervention (bias in performance), missing outcome data (bias in attrition), outcome measurement (bias in detection), selection of reported results (bias in reporting). The judgment of the domains was “low risk”, “some concerns” and “high risk”. In addition, the three judgment options were available for all risk biases.

## Results

### Literature search results

**Fig. 1:**
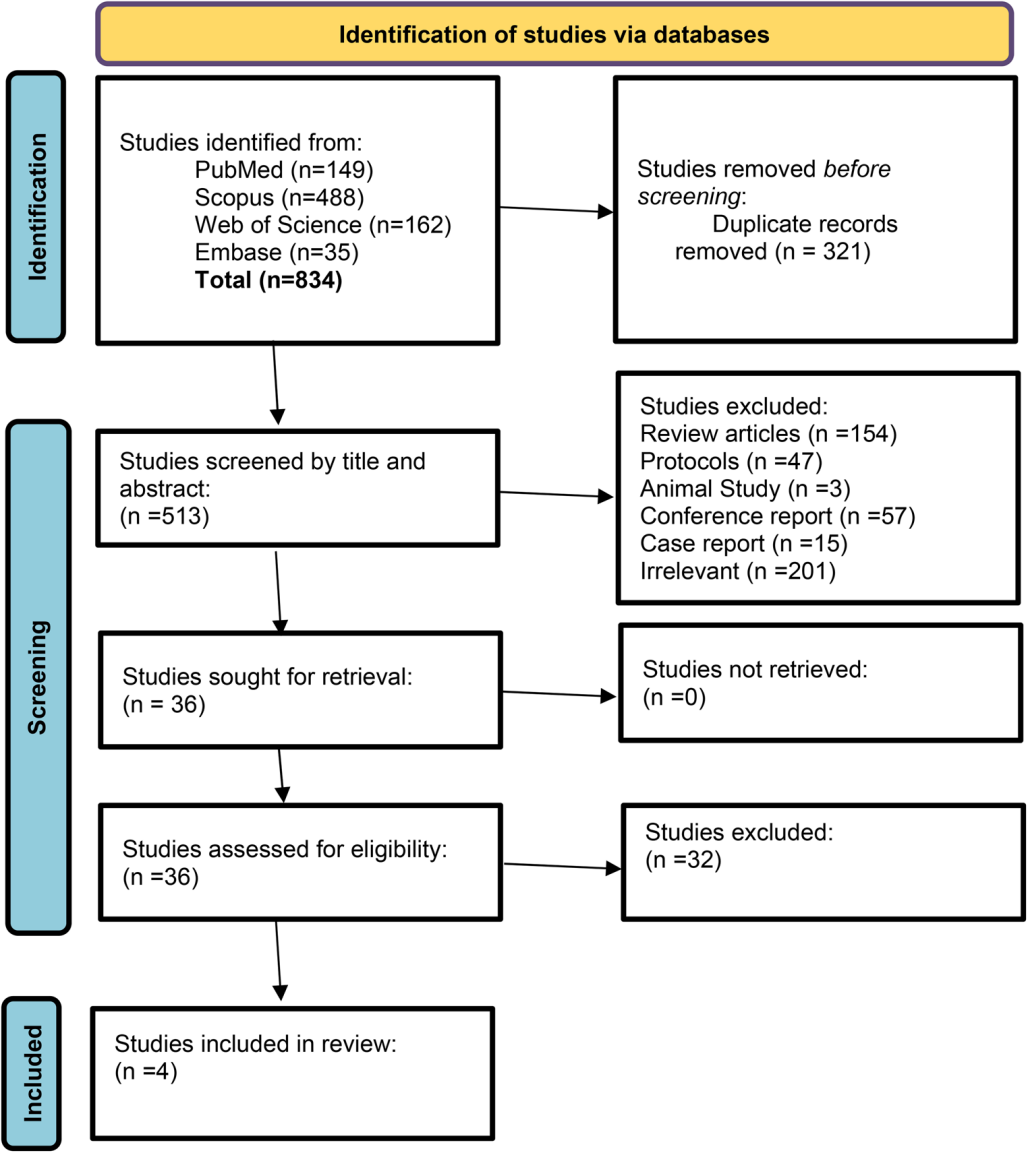
PRISMA flow chart.

Literature screening process is illustrated in the PRISMA flow diagram (Fig. 1). A Total of 834 unique articles were identified throughout the initial search. A total of 321 duplicates were removed, leaving 513 obtained records of which 477 articles were excluded after title and abstract screening and 32 articles were excluded after full-text screening upon given exclusion criteria. 4 RCTs^1–4^ were included in the systematic review, and they were also eligible for meta-analysis.

A total of 1401 patients were incorporated in the included studies. All cohort complained of axillary hyperhidrosis for which chosen option was GBP. Analyses were conducted comparing GBP to the control group or vehicle group.

### Characteristics of the included studies

Our study included 4 randomized controlled trials of 1401 patients receiving GBP compared to placebo group. (Baseline characteristics and the summary of the included studies are shown in (Tables 1 and 2).

**Table 1:** Summary of the included studies.

Study ID	Study design	Country	Drug under investigation	Active intervention	Comparator	Follow up duration	Aim of the study	Conclusion	Main inclusion criteria
**Abels**,** 2021**	randomized controlled trial	Germany Sweden Denmark United Kingdom Hungary	GPB	1% topical GPB cream	Placebo	4 weeks	To evaluate the effectiveness, safety, and acceptability of a topical GPB 1% cream treatment given to a patient for four weeks.	For patients with PAHH, GPB 1% cream may offer a novel, effective therapeutic alternative with a strong safety record. Phase IIIb, the long-term open-label portion, is still active.	Hyperhidrosis Disease Severity Scale (HDSS) score of 3 or 4, and with resting axillary sweat production in each axilla of > 50 mg in 5 min.
**Dee Anna Glaser**,**2018**	Randomized Vehicle-controlled trial	united states	GPB	2.40% topical GPB cream	vehicle	4 weeks	Examine GT’s effectiveness and safety in treating primary axillary hyperhidrosis.	When administered topically, GT was well tolerated in patients with primary axillary hyperhidrosis and decreased sweat production as evaluated gravimetrically and the severity of sweating as assessed by ASDD-Item 2 over a 4-week period.	at least 50 mg within 5 min in each axilla (HDSS) grade 3 or 4
**Hiroo Yokozeki1**,**2021**	Randomized Vehicle-controlled trial	Japan	GPB	3.75–2.5 topical GPB cream	Vehicle	4 weeks	assess the safety and effectiveness of GPB for Japanese patients with primary axillary hyperhidrosis.	For primary axillary hyperhidrosis, topical GT 3.75% and GT 2.5% cloths were used for four weeks. This led to a statistically significant and clinically substantial decrease	≥ 9 years of age who had primary axillary hyperhidrosis for at least 6 months, had sweat production of at least 50 mg/5 min for each axilla, and had a Hyperhidrosis Disease Severity Scale (HDSS) of 3 or 4
**M.Y. Hyun**,**2014**	randomized controlled trial	Korea	GPB	2% topical GPB cream	Placebo	nine successive days	To assess the topical glycopyrrolate’s antiperspirant effectiveness and safety on facial hyperhidrosis at predetermined posttreatment intervals	Applying topical glycopyrrolate seems to be a safe and very efficient way to stop excessive facial sweating.	100 mg of sweat for 20 min on each side of the forehead score 3 on Hyperhidrosis Disease Severity Scale (HDSS)

*GPB* glycopyrronium, *HDSS* Hyperhidrosis Disease Severity Scale, *ASDD* Axillary Sweating Daily Diary.

**Table 2:** Baseline characteristics of enrolled patients in the included studies.

Study ID	Age (Years)		Gender (Male)		Body weight		White		Black		Asian		ASDD/ASDD-C Item 2 score		DLQIc		CDLQId		HDSS for primary axillary hyperhidrosis Grade 3		HDSS for primary axillary hyperhidrosis Grade 4		Sweat production (mg/5 min),	
	Intervention	Comparator	Intervention	Comparator	Intervention	Comparator	Intervention	Comparator	Intervention	Comparator	Intervention	Comparator	Intervention	Comparator	Intervention	Comparator	Intervention	Comparator	Intervention	Comparator	Intervention	Comparator	Intervention	Comparator
Abels 2021	41.5 ± 35.43	41.5 ± 35.45	44 (51%)	43 (51%)	25.5 ± 2.267	25 ± 2.083	86(99%)	81 (96%)	1 (1%)	-	-	2 (2)		-	14 ± 5	15 ± 4.67	-	-	3 ± 0.333	4 ± 0.167	-	-		
Yokozeki 2021	38.45 ± 11.5	37.7 ± 10.9	132 (40.12%)	71 (43.03%)	60.77 ± 78.78	61.3 ± 12.1	-	-	-	-	-	NA	6.05 ± 1.65	5.9 ± 1.7	7.44 ± 5.61	6.7 ± 5.2	-	1.5 ± 2.1	125.06 ± 75.9	126 ± 76.4	39.5 ± 23.97	39 ± 24.2	116.48 ± 72.1	110.4 ± 72.1
Glaser 2018	32.3 ± 11	33.4 ± 12.2	212 (45.8%)	114 (48.7%)	27.5 ± 5.4	27.8 ± 5.2	374(80.8%)	196(83.8%)	59 (12.7)	30 (12.8)	5 (1.1)	0%	7.3 ± 1.6	7.2 ± 1.6	11.9 ± 6.1	10.6 ± 5.9	9.9 ± 5.5	8.5 ± 5.6	227 (59.8)	155 (66.2)	186 (40.2)	78 (33.3)	172.5 ± 215.7	176.2 ± 161.9

### Risk of bias and quality assessment

**Fig. 2:**
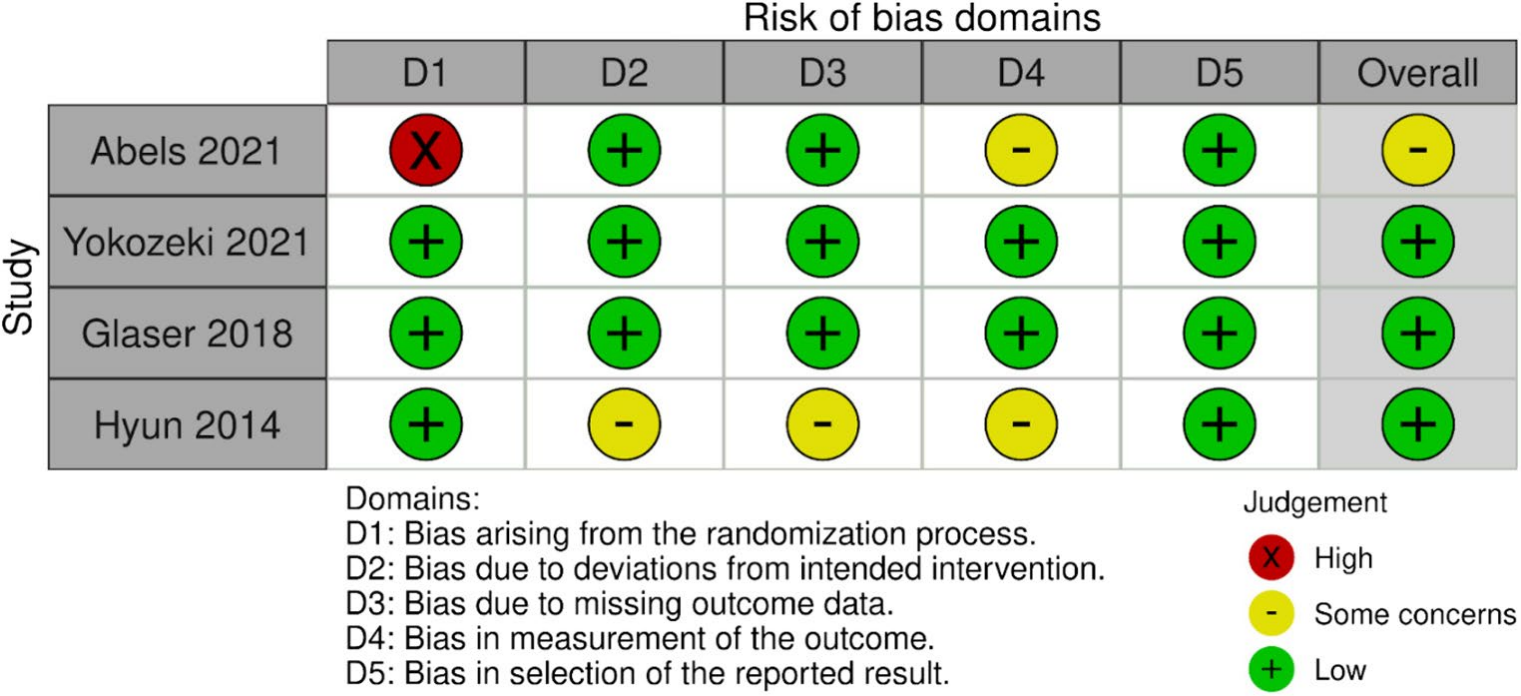
Summary of assessment of risk of bias.

Only Abels et al. 2021^1^ showed some concerns in the RoB-2 assessment and the other three RCTs showed low risk of bias (Fig. 2).

### Efficacy outcomes

Four studies measuring the effect of GBP for treatment of axillary hyperhidrosis were included. All studies used GBP topical cream with concentration/dose range of (1 − 3.75%).

#### HDSS responders

A subgroup analysis was done in which “assessing HDSS responders at day 15” showed a statistically significant difference in the overall effect in favor of GBP group (RR = 2.71, 95% CI [2.02 to 3.63], *p* < 0.00001); A random-effect analysis showed moderate heterogeneity among the included studies (*P* = 0.13, I2 = 47%).

There was a statistically significant difference in the overall effect on performing a subgroup analysis for “HDSS responders measured at day 29” which was in the favor of GBP group. (RR = 2.15, 95% CI [1.80 to 2.56], *p* < 0.00001); A random-effect analysis was not statistically significant among the included studies (*P* = 0.25, I2 = 26%).

**Fig. 3:**
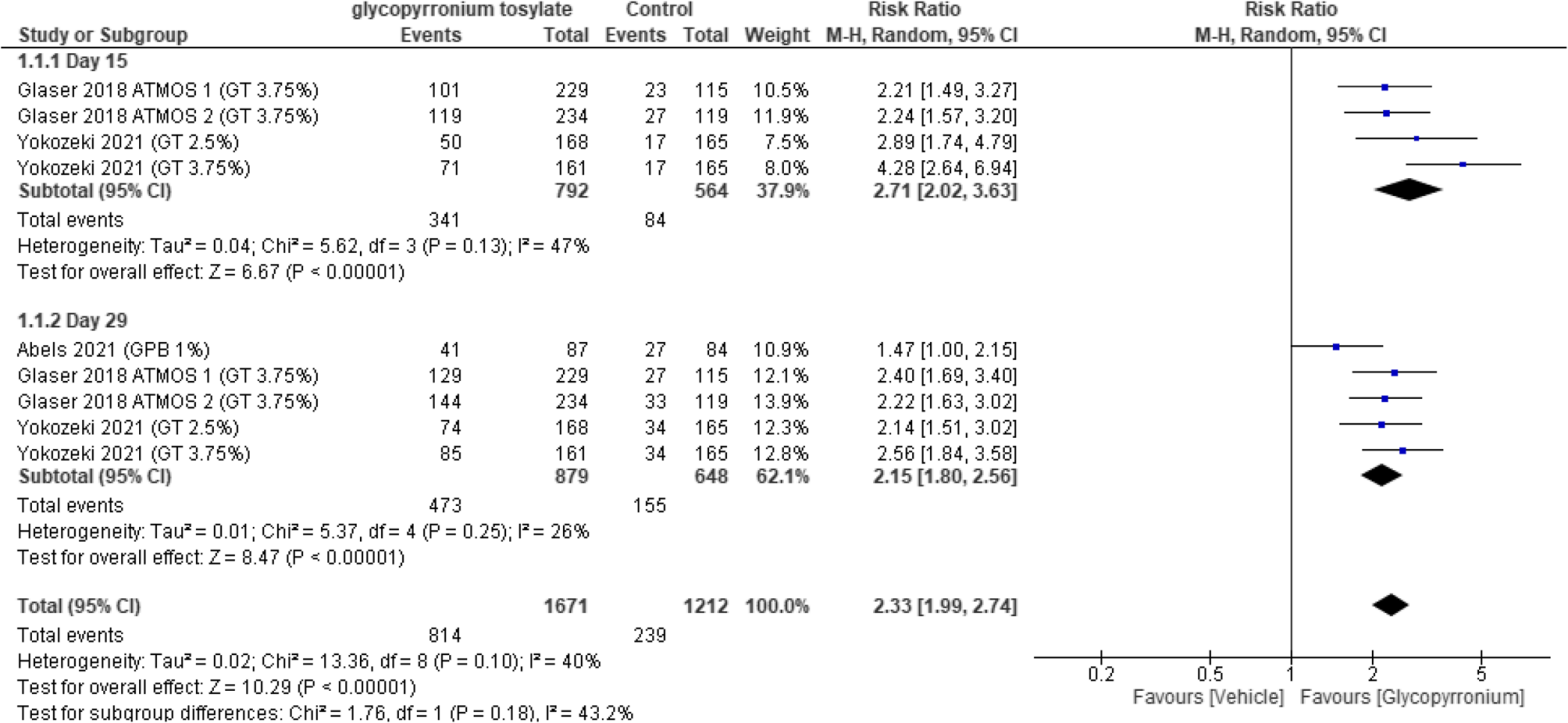
Forest plot for HDSS responders.

The pooled analysis results showed an overall statistically significant difference HDSS responders in favour of GBP group (RR = 2.33, 95% CI [1.99 to 2.74], *p* < 0.00001); and the pooled results were homogenous (*p* = 0.10, I2 = 40%) (Fig. 3).

#### Proportion of ASDD/ASDD-C responders

Subgroup analysis was performed to assess “proportion of ASDD/ASDD-C responders” at day 15 in which it showed a statistically significant increase (RR = 3.57, 95% CI [2.22 to 5.76], *p* < 0.00001)

And on assessing the proportion of ASDD/ASDD-C responders “at day 29 a sub-analysis revealed a statistically significant difference (RR = 2.76, 95% CI [1.90 to 4.01], p < 0.00001).

**Fig. 4:**
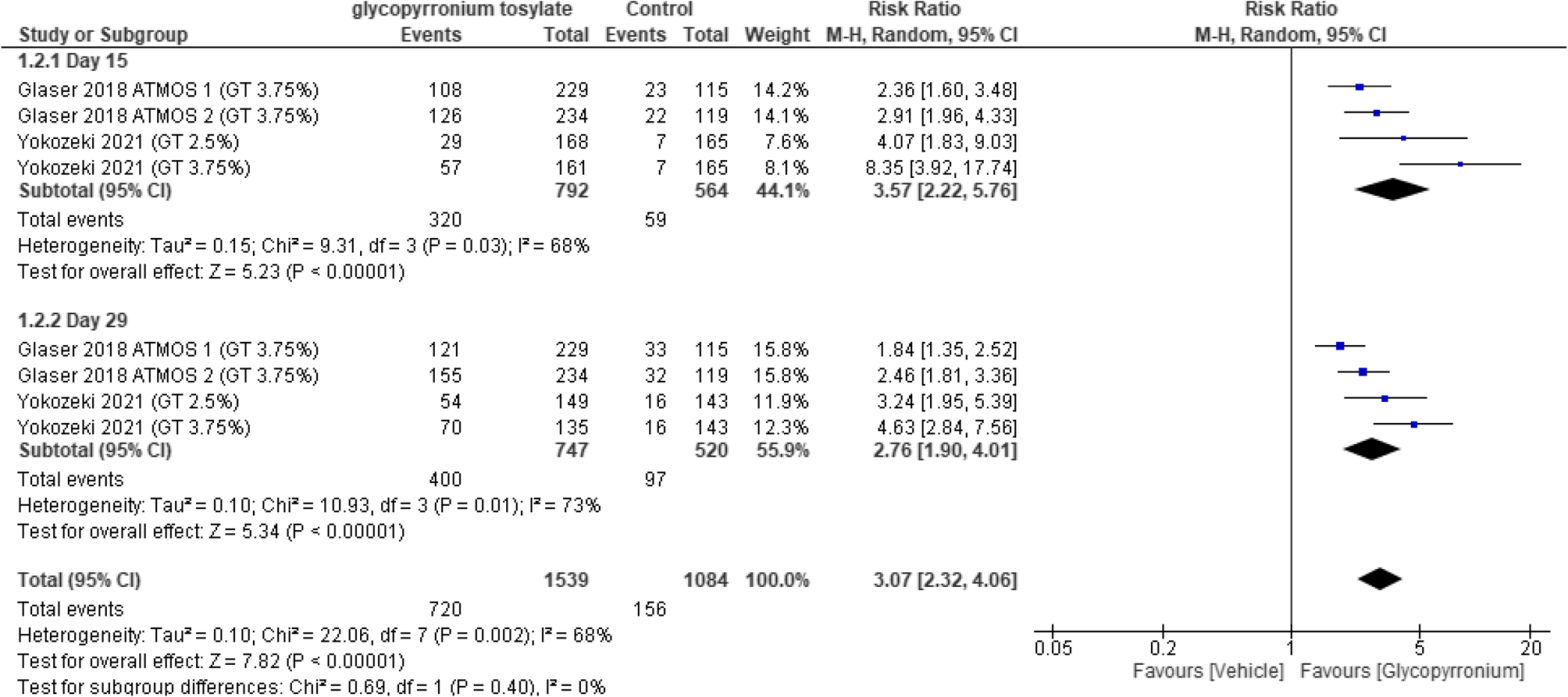
Forest plot for Proportion of ASDD/ASDD-C responders.

Pooled results for the proportion of ASDD/ASDD-C responders were statistically significant in favor of GBP group (RR = 3.07, 95% CI [ 2.32 to 4.06], *p* < 0.00001); All results showed heterogeneity (*P* = 0.002, I2 = 68%) (Fig. 4).

#### Sweat production (SP) responder rate

**Fig. 5:**
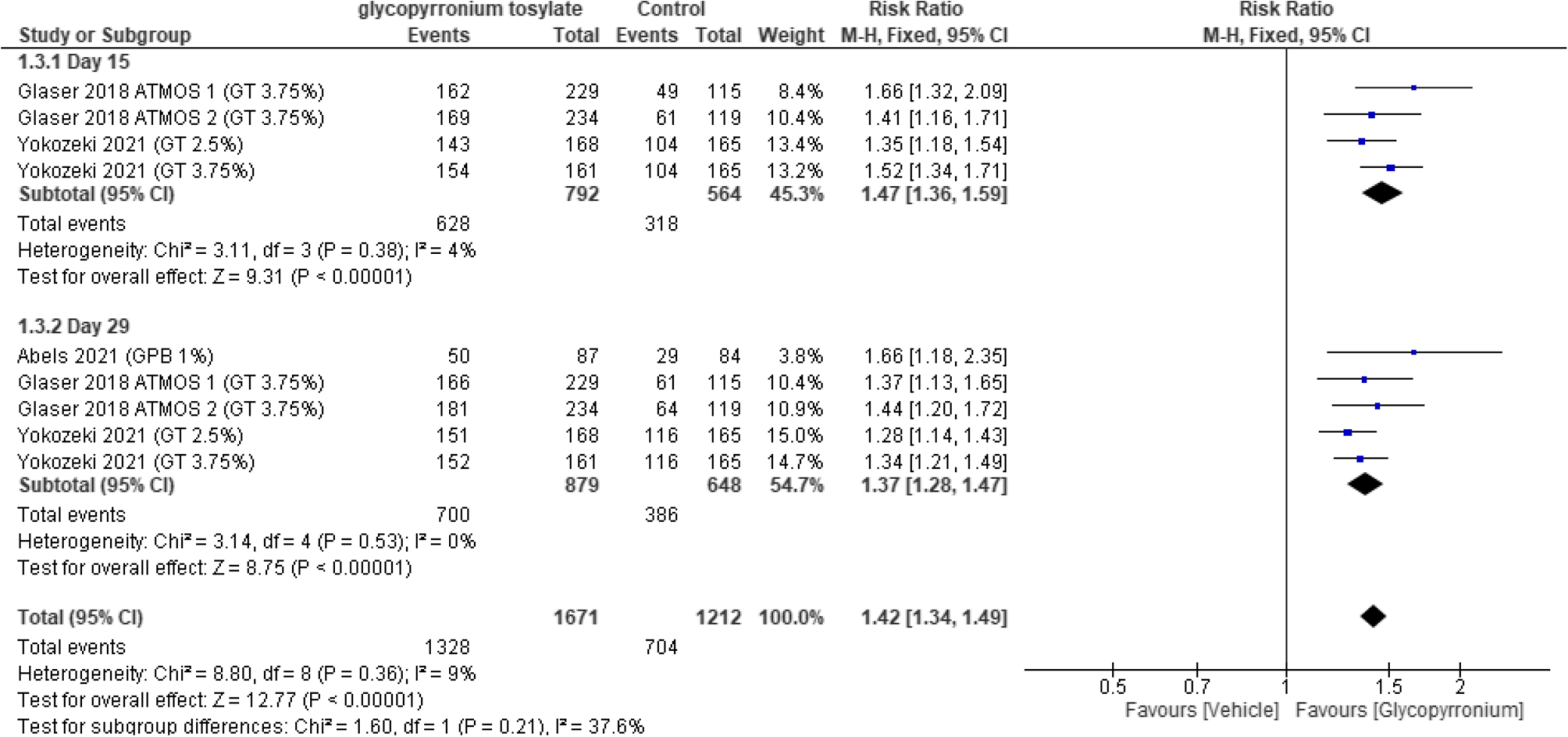
Forest plot for Sweat production (SP) responder rate.

According to SP response rate at day 15, a statistically significant difference was observed (RR = 1.47, 95% CI [1.36 to 1.59], *P* < 0.00001) and it was all for GBP group. Furthermore, on a performed sub-analysis on day 29, there was a statistically significant difference also supporting GBP (RR = 1.37, 95% CI [1.28 to 1.47]; *P* < 0.00001). On pooling, there was a statistically significant difference in the overall response favoring GBP group (RR = 1.42, 95% CI [1.34 to 1.49]; *P* < 0.00001) and showed homogeneity (*P* = 0.36, I2 = 9%) (Fig. 5).

#### HDSS

**Fig. 6:**
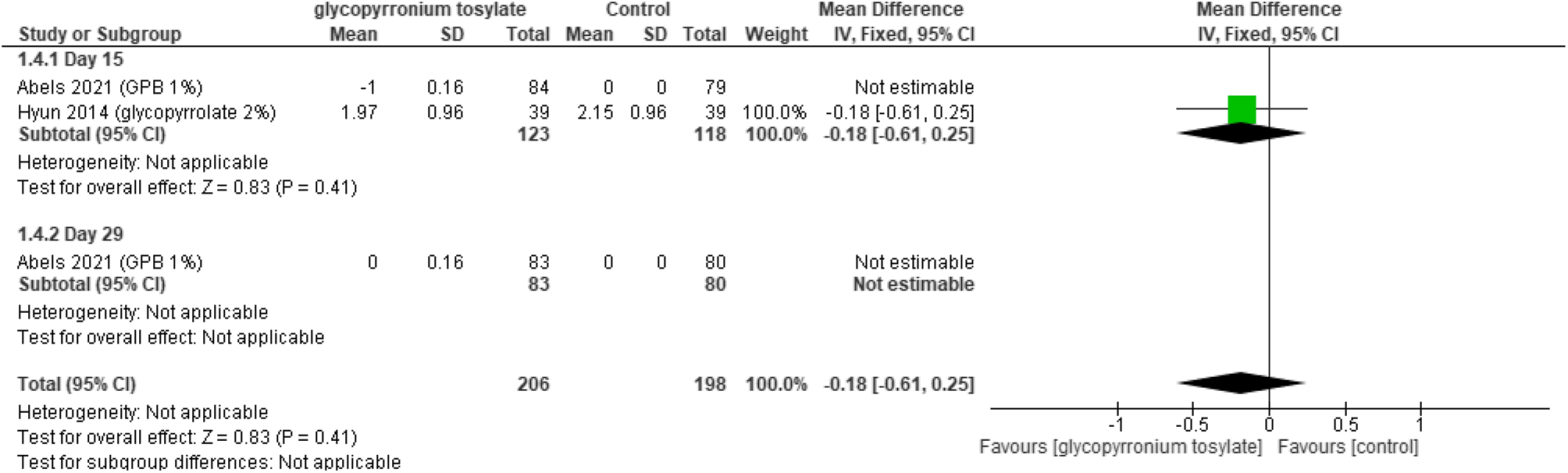
Forest plot for HDSS.

The overall mean difference between the control group and the GBP did not favor either of the two groups (MD = -0.18, 95% CI [-0.61 to 0.25], *P* = 0.4), heterogeneity was not applicable (Fig. 6).

#### Dermatology life quality index (DLQI)

**Fig. 7:**
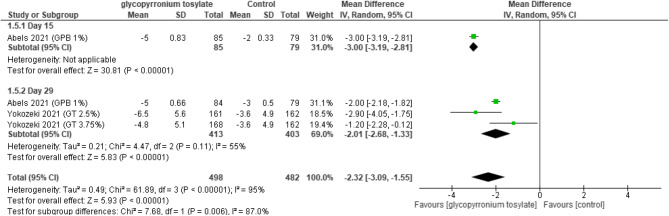
Forest plot for Dermatology life quality index (DLQI).

A sub-group analysis was performed over one study evaluating disease severity scale that hence showed a statistically significant decrease towards GBP group (MD = -3.00, 95% CI [-3.19 to -2.81], *p* < 0.00001), whereas in two studies evaluated at “day 29” statistically significant decrease was also noted (MD = -2.01, 95% [-2.68 to -1.33], *p* < 0.00001). And on pooling results, there was an overall statistically significant decrease in GBP group (MD = -2.32, 95% CI [-3.09, -1.55], *P* < 0.00001); heterogeneity was detected among groups (*P* < 0.00001, 95%) (Fig. 7).

#### Sweat production

**Fig. 8:**
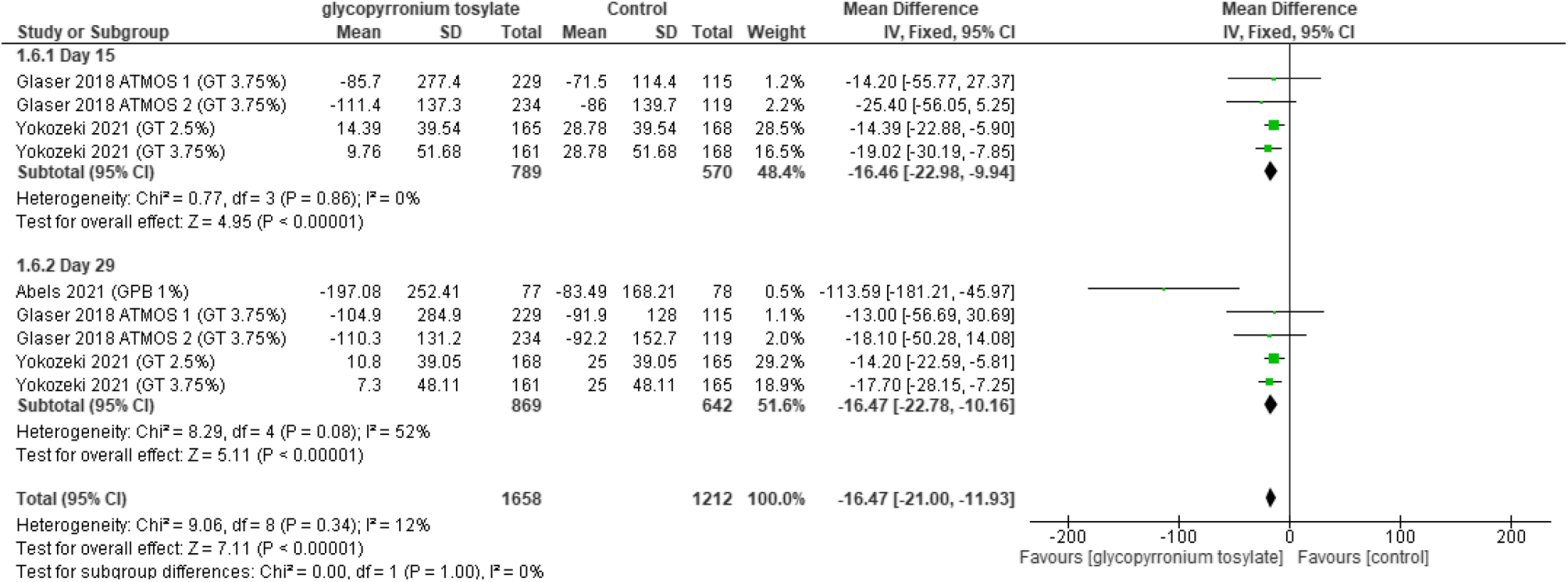
Forest plot for Sweat production.

Sweat production was set to be “assessed at day 15” and there was a statistically significant decrease noted favoring GBP (MD = -16.46, 95% CI [-22.98 to -9.94]; *P* < 0.00001). Meanwhile it was also assessed “at day 29” which also revealed a statistically significant decrease favoring GBP (MD = -16.47, 95% CI [-22.78 to -10.16]; *p* < 0.00001). Pooled analysis is suggestive of statistical decrease in the overall effect (MD = -16.47, 95% CI [-21.00 to -11.93]; *p* < 0.00001) and the pooled results were homogenous (*P* = 0.34, I2 = 12%) (Fig. 8).

#### Safety outcomes

**Fig. 9:**
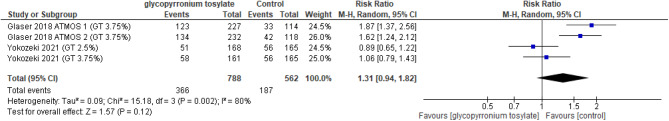
Forest plot for TEAEs.

**Fig. 10:**
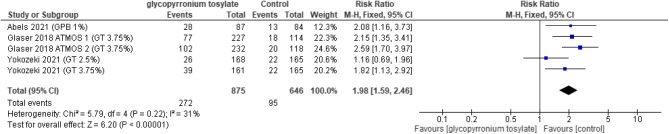
Forest plot for drug related TEAEs.

**Fig. 11:**
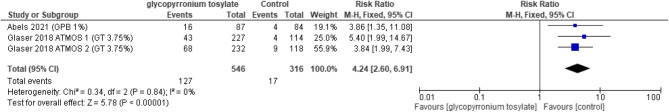
Forest plot for dry mouth.

**Fig. 12:**
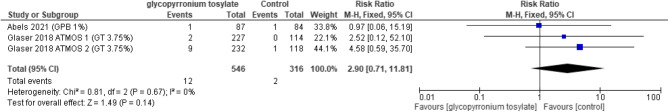
Forest plot for dry eye.

**Fig. 13:**
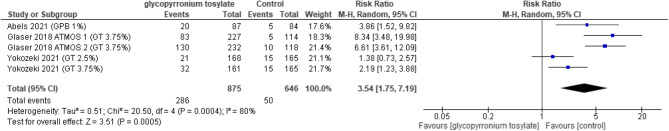
Forest plot for anticholinergic adverse events.

**Fig. 14:**
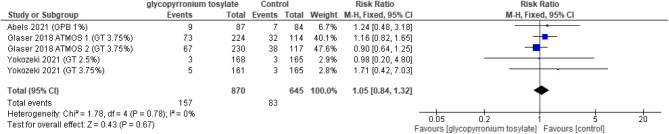
Forest plot for local skin reaction.

There was no statistically significant increase between the GBP group and the placebo group in the overall effect of adverse events (TEAEs) (RR = 1.31, 95% CI [0.94 to 1.82]; *P* = 0.12), with significant heterogeneity (*P* = 0.002, I2 = 80%) (Fig. 9). The overall risk ratio favored the control group in the drug related adverse events (RR = 1.98, 95% CI [1.59 to 2.46]; *P* < 0.00001), with no significant heterogeneity detected (*P* = 0.22, I2 = 31%) (Fig. 10). In dry mouth the overall risk ratio also favored the control (RR = 4.24, 95% CI [2.60 to 6.91]; *P* < 0.00001), and homogenous (*P* = 0.84, I2 = 0%) (Fig. 11). Regarding dry eyes there was no statistically significant difference between the two groups (RR = 2.90, 95% CI [ 0.71 to 11.81]; *p* = 0.14) with no heterogeneity (*P* = 0.67, I2 = 0%) (Fig. 12). The pooled results in anticholinergic adverse events favored the control group (RR = 3.54, 95% CI [1.75 to 7.19]; *P* = 0.0005), as heterogeneity was significant (*P* = 0.0004, I2 = 80%) (Fig. 13). In local skin reaction, no significant difference was detected between the two groups (RR = 1.05, 95% CI [0.84 to 1.32]; *P* = 0.67), which showed no heterogeneity (*P* = 0.78, I2 = 0%) (Fig. 14).

## Discussion

Hyperhidrosis create significant challenges to quality of life; hence, it is essential to investigate new treatment choices for affected patients. In order to address this issue, our study aimed to assess the efficacy and safety of topical glycopyrronium bromide as a treatment for primary hyperhidrosis. We analyzed the data from four randomized controlled trials (RCTs) to get valuable insights. Our findings indicate that glycopyrronium bromide has demonstrated effectiveness, showing favorable outcomes in patients with primary axillary hyperhidrosis.

Our meta-analysis revealed that the use of topical glycopyrronium bromide significantly reduced sweat production. In addition, HDSS Responders Subgroup analysis on both day 15^3,4^and day 29^1,3,4^demonstrated consistent and significant improvements favoring the glycopyrronium bromide group, reinforcing the efficacy of this treatment. This is further supported by a clinical trial conducted by Szeimies et al. that studied the long-term efficacy of 1% glycopyrronium bromide cream in patients with a severe form of primary axillary hyperhidrosis which found significant reductions in sweat production. Also, HDSS responders were significant in weeks 28, 52, and 72^5^. In patients with hyperhidrosis, topical glycopyrronium bromide is effective and improves management of symptoms; these results demonstrate the need for further studies to optimize treatment protocols.

Our analysis showed a significant increase in the proportion of Axillary Sweating Daily Diary (ASDD)/ASDD-C responders among patients using glycopyrronium bromide^3,4^. Additionally, the Dermatology Life Quality Index (DLQI) was significantly improved^1,4^. These findings are consistent with the results of the trial conducted by Szeimies et al., which shows improvement in quality of life as assessed by DLQI and Hyperhidrosis Quality of Life Index (HidroQoL©). These improvements strongly support the efficacy of glycopyrronium bromide cream over the entire study period^5^. These results are particularly promising as they indicate not only a physiological improvement but also a positive impact on the overall quality of life for individuals suffering from hyperhidrosis.

Our analysis showed a decrease in sweat production as indicated by both the Sweat Production (SP) Responder Rate and direct evaluation of sweat production. This highlights the potential of glycopyrronium bromide to treat the main symptom of hyperhidrosis effectively^1,3,4^. The degree of decrease in sweat production was significant, further emphasizing the effectiveness of this treatment.

Despite glycopyrronium bromide’s evident effectiveness, it remains crucial to investigate any associated health and safety concerns thoroughly. Our analysis demonstrated a significant rise in drug-related adverse events, specifically dry mouth^1,3^, and anticholinergic adverse events. In the Szeimies et al. trial, only a few adverse drug reactions were observed, with dry mouth being the most common and expected anticholinergic reaction. The occurrence of dry mouth, a frequently observed anticholinergic adverse effect, was significantly higher in the group receiving glycopyrronium bromide^1,3^. In clinical research conducted by Pariser et al., a comparison was made between topical glycopyrronium tosylate and oral glycopyrrolate. The results showed that there was limited absorption and a low risk of anticholinergic Adverse events with proper glycopyrronium tosylate administration. Adverse events may occur when it is administered incorrectly. It is important for patients to follow specific instructions, which include wiping each underarm once with the same cloth, washing hands with soap and water immediately after discarding the cloth, and avoiding eye contact with the drug to prevent blurred vision and temporary dilation of the pupils^21^Clinicians should carefully weigh the therapeutic advantages against the potential discomfort caused by adverse events. Given the significance of evaluating potential risks associated with cumulative exposure to anticholinergic drugs, this remains essential even when using a topical formulation. One of the main concerns with anticholinergic medications such as glycopyrronium bromide is their potential to cause cognitive impairment and other central nervous system side effects. Studies have shown that long-term use of anticholinergic drugs can lead to cognitive decline and an increased risk of developing conditions like dementia. Moreover, the presence of anticholinergic adverse events emphasizes the necessity for careful patient selection and personalized treatment strategies. Using a topical form of glycopyrronium bromide can be a good option for some patients, as it allows for targeted application to the affected area and may reduce the systemic absorption of the medication compared to oral forms. This can potentially lower the risk of systemic side effects associated with anticholinergic medications. Owing to the importance of considering the potential risks of cumulative exposure to anticholinergic drugs, even when using a topical form^22^.

Significantly, the absence of a significant increase in dry eye^1,3^and local skin reactions^1,3,4^ indicates that glycopyrronium bromide has a rather favorable safety profile in these aspects. Nevertheless, the general rise in treatment emergent adverse events (TEAE) compared to the control group warrants an accurate assessment of the risk-benefit ratio when choosing glycopyrronium bromide as a therapeutic choice. The specific types of TEAEs observed and their potential impact on patient health and quality of life should be considered in this assessment.

This systematic review and meta-analysis is one of the first to review the efficacy and safety of topical glycopyrronium bromide. The inclusion of randomized controlled trials (RCTs) only enhances the strength of our findings, which provides significant insight into the treatment’s effectiveness.

Despite these strengths, it is necessary to address the limitations. The limited numbers of included studies, only four randomized controlled trials (RCTs) were found throughout the screening process, which underlines the lack of experimental studies assessing the efficacy of topical glycopyrronium bromide in treating hyperhidrosis. Furthermore, the presence of heterogeneity in certain safety outcomes established an additional limitation of this study. As well as no publication bias analysis was done due to the small number of included RCTs.

Subsequent investigations should aim for consistent records and extended monitoring to assess the sustained effectiveness and safety of topical glycopyrronium bromide more effectively. To successfully integrate this potential therapy into ordinary clinical practice and develop usage guidelines, it is necessary to conduct randomized controlled trials (RCTs) with larger sample sizes and a long-term follow-up period.

In conclusion, topical glycopyrronium bromide is an effective treatment for axillary hyperhidrosis, associated with significant sweat reduction and improvement of symptoms. However, it is important to consider the potential risks associated with the medication’s long-term use. The observed increase in certain adverse events necessitates careful consideration in clinical decision-making, particularly when it comes to cumulative exposure to anticholinergic drugs. To solidify these findings and optimize the use of topical glycopyrronium bromide in managing primary hyperhidrosis patients, we recommend further research and long-term studies.

## Data Availability

The datasets used and/or analyzed during the current study are available from the corresponding author on reasonable request.
